# Arterial bleeding during EUS-guided pseudocyst drainage stopped by placement of a covered self-expandable metal stent

**DOI:** 10.1186/1471-230X-13-93

**Published:** 2013-05-24

**Authors:** Adrian Săftoiu, Lidia Ciobanu, Andrada Seicean, Marcel Tantău

**Affiliations:** 1 Research Center of Gastroenterology and Hepatology Craiova, University of Medicine and Pharmacy Craiova, Romania; 2 Gastrointestinal Unit, Faculty of Health and Medical Sciences, Copenhagen University, Copenhagen, Denmark; 3 Regional Institute of Gastroenterology and Hepatology University of Medicine and Pharmacy Cluj-Napoca, Romania

**Keywords:** Endoscopic ultrasound, Pancreatic pseudocyst, Upper gastrointestinal bleeding, Portal hypertension

## Abstract

**Background:**

Hemorrhagic complications during EUS-guided pseudocyst drainage can occur, because the vessels on the internal wall of the pseudocyst might be compressed by the fluid and thus not visible on color Doppler or even power Doppler EUS.

**Case presentation:**

We report a case of an immediate internal spurting arterial bleeding precipitated during EUS-guided pseudocyst drainage which stopped instantaneously by placement of a double flanged covered self-expandable metal stent through mechanical hemostasis.

**Conclusion:**

In an unusual situation of bleeding from collateral circulation near the pseudocyst wall during pseudocyst drainage, the placement of an expandable metal stent proved to be useful.

## Background

Endoscopic ultrasound (EUS)-guided pseudocyst drainage is nowadays a routine procedure performed in tertiary centers, with minimal morbidity and mortality. Initial procedures based on one-step EUS-guidance were developed almost 15 years ago and they still represent the standard of care in most advanced GI units [1,2]. During the years, EUS-guided procedures for gastric or duodenal transmural drainage of pancreatic pseudocysts benefitted from a certain number of technical improvements, although the technique still has a long learning curve [3,4]. Despite the increased safety of transmural puncture based on the avoidance of major vessels with increased use of EUS Doppler techniques, there are still immediate complications consisting mainly of perforations and bleeding in about 1-2% of cases [5,6]. Nevertheless, if there is a careful selection of the patients, the EUS-guided drainage procedure is considered safer as compared with surgical cyst-gastrostomy [7]. The case report describes an immediate spurting arterial bleeding precipitated during EUS-guided pseudocyst drainage which stopped instantaneously by placement of a double flanged covered self-expandable metal stent.

## Case presentation

We present the case of a 41 years old male with a long standing history of ethanol consumption. His past medical history was significant for pancreatic disorders: he had two episodes of acute pancreatitis induced by ethanol consumption. Four weeks before the drainage procedure, he presented in the emergency department presenting with intense upper abdominal pain, nausea and vomiting, induced again by heavy ethanol consumption. Biological markers revealed elevated levels of blood and urine amylases (466 IU/L and 2397 IU/L) and leucocytosis. An emergency transabdominal ultrasound identified a large pseudocyst located near the pancreatic tail, confirmed by the contrast-enhanced CT scan. No other fluid collection or necrotic areas were revealed. Nevertheles, the CT scan indicated the presence of the splenic vein thrombosis and the collaterals near the pancreatics tail region, as well as in the gastric wall.

**Figure 1:**
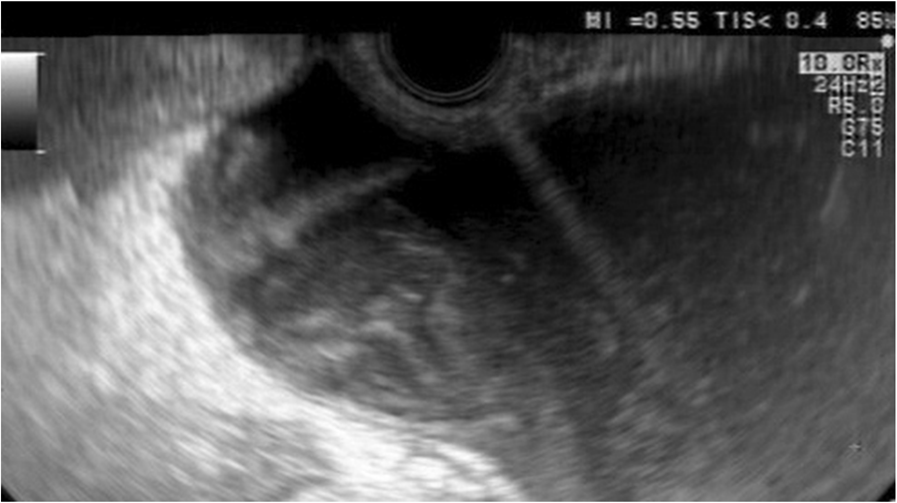
Significant spurting (pulsatile) arterial bleeding originating from the puncture level inside the pseudocyst.

As the initial therapeutic option, we took into account the possibility of a transcutaneous ultrasound-guided approach or an endoscopic approach by placing a stent using EUS-guidance from the stomach. As an important collateral circulation developed as a consequence of splenic vein thrombosis, after the inform consent was signed, our choice was endoscopic stenting using an EUS-guided procedure with avoidance of major vessels through the use of Doppler techniques. We used a therapeutic EUS scope with a large 3.8 mm channel (Olympus GIF-UCT 140, Olympus, Tokyo, Japan) coupled with the corresponding ultrasound system (Aloka Alpha10, Aloka, Tokyo, Japan). The pseudocyst was visualised from the smaller gastric curvature and an area thought to be devoid of major vessels was carefully selected by the use of color and power Doppler. Under EUS-guidance the pseudocyst was punctured using the special device with a trocar and cutting blade (Navix, Xlumena, Mountain View, California, USA). During the passage of the cutting blade through the gastric wall a significant spurting (pulsatile) arterial bleeding started from the puncture level inside the pseudocyst, being easily visible on the gray-scale mode (Figure 1, Additional file 1: Movie S1), with minimal intragastric bleeding. A 0.035″ hydrophilic guidewire (Cook, Limerick, Ireland) was placed through the 19 G trocar and coiled deeply inside the pseudocyst. The procedure has been continued with both EUS-guidance, but also intermittent radiological check-ups of the guidewire position inside the pseudocyst.

**Figure 2:**
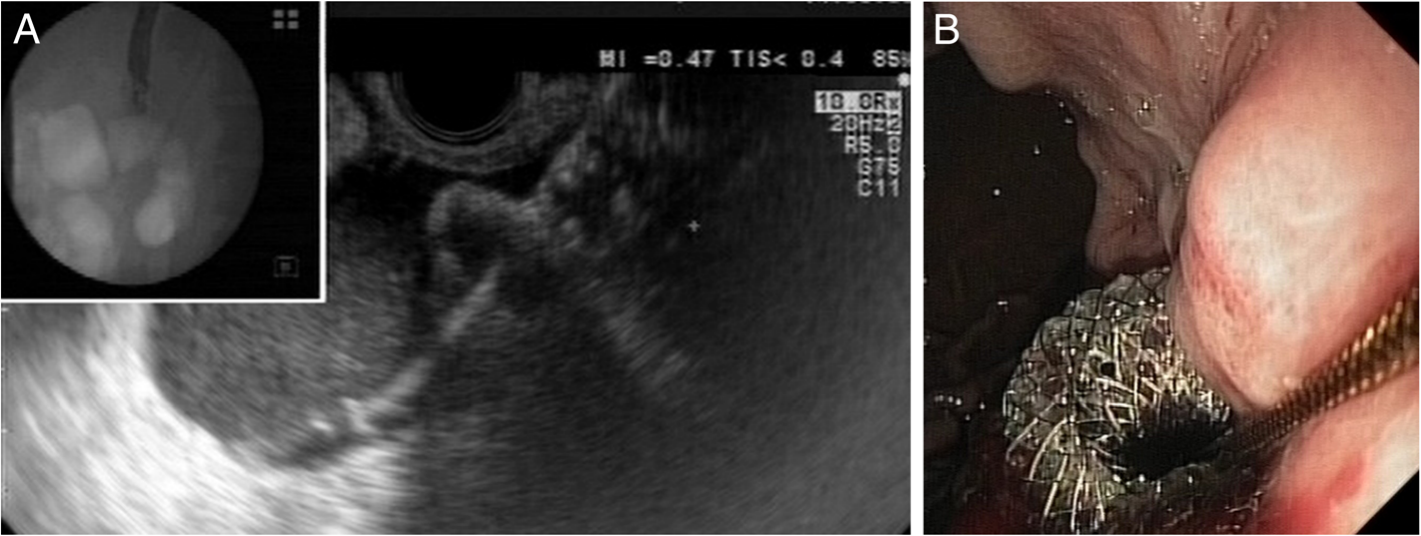
**Deployment of the stent under EUS and endoscopic guidance. A**. Deployment of the distal part of the stent under combined EUS and radiological guidance. **B**. Deployment of the proximal part of the stent under direct endoscopic guidance.

A special double flanged covered expandable stent of 10 mm in the saddle length (between the two flanges) and 10 mm in diameter when expanded (Axios, Xlumena, Mountain View, California, USA) was then uploaded on the guidwire. The stent is collapsed into a 10.8 Fr delivery catheter system which adapts into the therapeutic channel of an EUS scope and then expands separately for the distal and proximal flanges, without the ability of being collapsed into the delivery catheter. The stent was then deployed from the distal part under EUS and radiological guidance (Figure 2A-B), followed by deployment of the proximal part under direct endoscopic guidance, using special manufacturers instructions and the steps provided on the delivery system handle. As a consequence of stent deployment and pressure elicited on the gastric and pseudocyst wall at the level of the fistula created during the EUS procedure, the bleeding stopped instantaneously. The patient was then treated with a proton pump inhibitor and large spectrum antibiotics (cefuroxime and metronidazol) for five days, with fever of up to 38.3 degrees C, which decreased to normal after 24 hours.

After 4 weeks with ethanol abstinence, the patient returned for another episode of pain in the upper abdomen and fever, associated with leucocytosis, but normal amylasemia. Although, there were no collections visualised on ultrasound or contrast-enhanced CT scan, a decision to remove the stent endoscopically was taken. The stent was easily snare and taken out, with minimal bleding of the gastric wall which stopped after adrenaline injections (1 : 10 000) and argon plasma coagulation. The pain immediately and the patient was discharged. There were no episodes of bleeding or recurrent acute pancreatitis during a 6 months follow-up, and the pseudocyst did not recurred.

### Discussions

Bleeding during drainage of pancreatic pseudocysts is a severe complication as it might occur immediately [8] or delayed [9], leading to increased morbidity and even mortality. These are due to prolongation of the period and an increased transfusion rate, as well as because of the necessity of emergency surgical intervention. Moreover, the subgroup of patients with complications after the drainage of pancreatic pseudocysts are the most prone to an increased mortality. Hence, transcutaneous or endoscopic drainage of pancreatic pseudocysts were replaced by EUS-guided drainage procedures which decrease significantly the risk of bleeding [10]. Nevertheless, even with the use of real-time guidance during EUS, there are still hemorrhagic complications in less than 1-2% of cases, because the vessels on the internal wall of the pseudocyst might be compressed by the fluid and thus not visible on color Doppler or even power Doppler EUS. In our case, the sudden spurting arterial bleeding was most probably casused by the blade which extends in the tip of the 19G trocar, probably by damaging concealed vessels at the internal wall of the pseudocyst, which were not visible initially, but were probably decompressed during EUS-guided drainage, through initial aspiration of the fluid, but also during exchange of the EUS accesories which probably lead to leakage of the pseudocyst fluid.

Although single or multiple plastic stents, as well as nasocystic drainage catheters are used for EUS-guided pseudocyst drainage, expandable stents were also considered as viable alternatives as they keep open the tract created between the stomach and pseudoscyst cavity. This is beneficial especially for the cases with large, infected pseudocysts or pancreatic abscesses. Several articles already described the use of covered expandable metal stents for the drainage of pancreatic pseudocysts, having the advantage of a large diameter with a possible decrease in the reccurence rate [11]. There are however disadvantages which includ stent migration and difficulty of insertion for large stents [12]. Even surgical approaches were described, with the use of staplers applied transorally by minimal invasive NOTES procedures, after an initial EUS-guided approach [13]. For our patient we chose a novel metalic stent with double flanges, which have the intended advantage of preventing migration, but also induce apposition of the pseudocyst wall and gastric/duodenal wall, through the covered part, thus preventing leakage and decreasing the perforation risk [14,15]. However, due to the large diameter (10.8 Fr) of the delivery system, the insertion through the gastric and pseudocyst wall might be difficult if a previous dilation of the initial tract is not performed using baloon dilators and/or cautery devices (needle knife sphincterotome, hot needle wires or cystotomes). Even under real-time EUS-guidance with use of color Doppler techniques, the initial dilation of the tract before stent insertion can possibly cause significant bleding at the level of the digestive tract wall or pancreatic pseudocyst wall collaterals which might be compressed by the fluid inside the pseudocyst. Nevertheless, the advantage of a covered expandable stent is exactly the possibility of stopping the bleeding through mechanical hemostasis and this has been clearly demonstrated in our case report.

The use of metallic stents in order to control severe bleedings in the GI tract has been already described in several articles. Thus, a review of preliminary reports suggested that self-expandable covered metal stents might be considered useful and very effective to control refractory acute variceal bleeding, being an alternative to the temporary control obtained by Sengstaken-Blakemore baloon [16]. The stents used have a modified design (with atraumatic edges and retrieval loops with gold markers at both stent ends) which allows them to be extracted with a special system after a few days [17]. Covered self-expandable metallic stenting was also described to be useful in post-sphincterotomy bleeding [18,19] or other GI malignant tumors diffuse bleeding [20] or in case of the conventional endoscopic fluid collection drainage [21], which does not stop after usual endoscopic treatment (injection, coagulation, clipping, etc.). Our case presentation proved that the same approach is useful in pseudocyst bleeding that occurs during EUS-guided drainage.

## Conclusions

This case report shows a rare case of bleeding during the EUS drainage procedure of pancreatic pseudocyst at the level of the pseudocyst wall. The double flanged covered self-expandable metal stent proved its utility in such unusual cases with high risk for bleeding due to colateral circulation.

Written informed consent was obtained from the patient for publication of this Case report and any accompanying images. A copy of the written consent is available for review by the Editor of this journal.

## Abbreviations

EUS: Endoscopic ultrasound; CT scan: Computed tomography.

## Competing interests

The authors declare that they have no competing interests.

## Authors’ contributions

ASaftoiu did the EUS-guided pseudocyst drainage, LC, ASeicean and MT selected the patient for the drainage and followed him up. All authors have been involved in drafting the manuscript or revising it and have given final approval of the version to be published.

## Pre-publication history

The pre-publication history for this paper can be accessed here:

http://www.biomedcentral.com/1471-230X/13/93/prepub

## Supplementary Material

Additional file 1: Movie S1 Significant spurting (pulsatile) arterial bleeding originating from the puncture level inside the pseudocyst. Click here for file
